# Advances in artificial intelligence-based approaches to enhance dark field X-ray microscopy analysis

**DOI:** 10.1557/s43579-025-00860-4

**Published:** 2025-12-08

**Authors:** Brinthan Kanesalingam, Can Yildirim, Leora Dresselhaus-Marais

**Affiliations:** 1 Materials Science and Engineering, Stanford University, Stanford, CA 94305 USA; 2 SLAC National Accelerator Laboratory, Menlo Park, CA 94025 USA; 3 European Synchrotron Radiation Facility, 38043 Grenoble, France

**Keywords:** Dark field X-ray microscopy, Dislocation dynamics, Artificial intelligence, Physics informed AI, Dimension reduction

## Abstract

Dark field X-ray microscopy (DFXM) has emerged as a powerful technique for characterizing dislocations in bulk crystalline materials, whose high penetration depth and non-destructive evaluation offer unique advantages over traditional electron microscopy methods. The interpretation and analysis of the DFXM data presents significant challenges that have limited its broader adoption. Here we review our recent advances using artificial intelligence (AI) methods to enhance DFXM analysis, particularly focusing on dislocation characterization. We discuss the development of physics-informed AI approaches that combine theoretical understanding with data science techniques to enable both time-resolved dislocation dynamics studies and dimensional reduction of complex DFXM datasets. Our work demonstrates how semi-automated workflows, guided by dislocation theory and employing techniques such as wavelet transforms and Bayesian inference, can effectively track and analyze dislocation behavior across multiple time scales. These methodologies have been successfully applied to various materials science challenges, from studying thermally activated dislocation motion to characterizing dislocation networks. By presenting our works that integrate physics-based modeling into AI capabilities, we demonstrate how our and other works can extract new important quantitative dislocation data from DFXM measurements.

**Graphical abstract:**

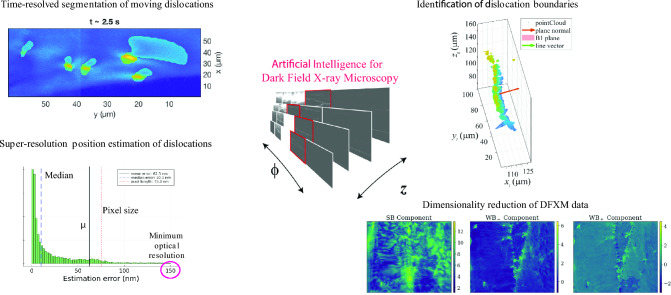

## Introduction

Dislocations play a critical role in the determination of material properties in various industries. In functional materials, dislocations contribute to the mechanisms driving phase transitions, such as those in ferroelectric materials used in non-volatile computer memory.^1,2^ In metallurgy, the motion of the dislocation and interactions govern the plasticity of the crystal, dictating the mechanical performance of the alloys under various loading conditions.^3^ In electronic and thermal applications, dislocations scatter charge carriers and phonons, influencing, respectively, electrical and thermal conductivity.^4^ Across all of these and many other applications, the control of materials requires us to be able to identify, measure, and understand how dislocations respond to their surroundings.

However, identifying dislocations and characterizing how dislocations give rise to macroscopic quantities has been a difficult task for nearly a century^5^ because dislocations span length scales from atoms to meters and timescales from single atomic vibrations of $\sim$ 100 femtoseconds to hours or years.^6^ Describing a population of dislocations in a crystal requires atomic information for full accuracy; the high-dimensional data this would necessitate at the meter-scale poses an unmanageable challenge even for today’s most powerful supercomputers because of the large volume of data.^7^ Therefore, the most crucial and challenging aspect of this field is to find ways to effectively reduce the dimensionality of this problem while retaining the appropriate physics so that, on the continuum scale, the relevant material behavior still accurately reflects reality.^8^ Of course, no single solution works for all properties, defects, and loading conditions; thus, we must establish new methods and tools to identify and characterize the dislocations.

As noted above, the characterization of dislocations has long been a challenging task, dating back to when they were first proposed in 1934 by Taylor,^9^ Orowan,^10^ and Polanyi.^11^ After 20 years of theorizing, direct observation of dislocations became possible in the 1950 s with the advent of transmission electron microscopy (TEM) by Hirsch *et al.*^12^ Today, TEM and atomic force microscopy (AFM) remain standard techniques for imaging dislocation cores at atomic resolution and mapping dislocation lines over hundreds of nanometers.^13,14^ More recently, time-resolved versions of these microscopes have offered key insights into dislocation patterning and behaviors.^15^ However, these techniques have a small field of view that is not necessarily representative of the billions of dislocations relevant to the statistical populations responsible for the properties.^16^ Additionally, these microscopes can resolve either the surface or through the thickness of very thin samples; this limitation introduces image forces that are known to wildly change the behaviors of dislocations, making their dynamics not necessarily representative of the bulk systems in most technologies.^17^ Consequently, many problems in dislocation dynamics have been studied primarily by models, with validation by low-dimensional datasets whose average values have no unique solution to constrain dislocation behaviors.^18^

Compared with electron microscopy techniques, X-ray techniques offer several unique advantages. Specifically, unlike electron microscopy techniques, X-ray techniques offer a high penetration depth in most materials, allowing non-destructive probing deep beneath their surface.^19^ X-ray topography has been widely used to image extended defects such as dislocations in single-crystal semiconductors.^20,21^ Both monochromatic and white beam modes enable large-area mapping based on the sensitivity of diffracted intensity to local lattice distortions. While near field topography offers strong defect contrast, it is limited in spatial resolution and angular resolution perpendicular to the diffraction vector, including 2$\theta$. These limitations have driven the development of techniques with improved structural and angular resolution. Moreover, with recent advances in 4th-generation synchrotron sources and X-ray free electron lasers (XFELs), existing techniques are no longer constrained by the challenges posed by brightness and spatial coherence during measurements.^22^ In addition, other advances in X-ray optics now enable X-ray lenses, apertures, and phase plates that were not previously available, allowing a wide range of opportunities for X-ray microscopy techniques.^23^

Dark field X-ray microscopy (DFXM) has been developed over the past 10 years to image deformations in crystalline materials using an X-ray objective lens that is placed along the diffracted beam.^24^ The resulting real space images have bright and dark features that encode information about a specific lattice spacing and orientation in a crystal. The information from these images is related to the strained heterogeneous regions within a single grain that arise from distortions (e.g., loading, phonons) or defects, with selectivity based on the symmetry of the strain field.^25^ DFXM is a full-field imaging technique capable of monitoring dynamic processes deep inside mm-sized samples. Some examples of such dynamic processes that can be captured thanks to the full-field nature of the technique include dislocation dynamics,^26^ recrystallization and grain growth in metals,^27,28^ and ferroelastic domain switching.^29^ DFXM typically uses a monochromatic X-ray beam, while a recently developed pink-beam mode utilizes a broader energy bandwidth enabling significantly faster data acquisition and enhanced time resolution for dynamic studies.^28^

**Fig. 1:**
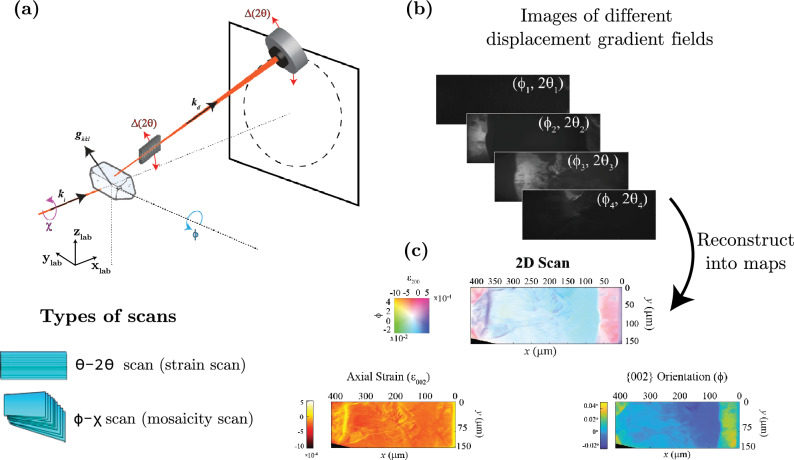
Detailed schematic diagram illustrating the scanning modalities in DFXM. (a) Represents a standard DFXM setup illustrating the scanning axes labeled as $\chi$, $\phi$, and $\Delta (2\theta )$. Here ${\textbf {k}}_{\text {i}}$, ${\textbf {k}}_{\text {d}}$, and ${\textbf {g}}_{hkl}$ represents average incident wavevector, average diffracted wavevector, and local scattering vector from *hkl* planes, respectively. (b) Presents a selection of representative sample images acquired for varying values of $\phi$ and $2\theta$. These images reveal different displacement gradient fields. (c) Provides examples of the types of reconstructed maps, such as the axial strain map and orientation maps, which are derived from the images shown in (b).

As shown in Fig. 1, DFXM experiments often use a 1D prefocussing lens to image with a linear beam that illuminates a sheet (i.e., the “observation plane” in the field of microscopy) inside the crystal that limits the integration volume (or gauge volume) described by the image.^30^ A typical DFXM experiment gathers “rocking-curve scans” that consist of a series of images as the sample rotates along different axes, referred to by the Eulerian cradle goniometer system as $\chi , \phi , \omega$, and/or the detector and lens are rotated commensurately in an arc to scan along 2$\theta$ and azimuthal angle $\eta$. The resulting data often include thousands of images that describe the different orientations and strain states in the material described by each pixel of the images (i.e., a voxel).^30,31^ After the data are collected, the images may be reconstructed into the orientational distribution for each pixel and fitted to a distribution function (Gaussian or Lorentzian) for which the mean and variance are saved for each motor scanned. The resulting “Center of Mass” (COM) plots describe the peak of that Gaussian for the orientation and/or strain most strongly represented for each measured pixel.^32,33^ Plots of the variance can offer some insight into how effectively the COM describes the data (i.e., indicates regions for which a multimodal distribution is likely).

Although only two dedicated beamlines offer DFXM to date,^34,35^ it has been extended to X-ray free electron lasers (XFELs) and is being developed at other beamlines.^36^ Since being first demonstrated,^24^ DFXM has solved critical challenges in ferroelectrics,^37^ phase transitions,^38^ thermal fatigue in power electronics,^39^ dislocation–dendrite interaction in batteries,^40^ biomaterials,^41^ operando performance of infrared detectors,^42^ and other fields. However, DFXM’s applications are still limited today by the challenges of interpreting and analyzing the large datasets, as well as the difficulty in quickly calibrating the microscope to the appropriate levels of precision. Each of the original DFXM experiments used home-built code to perform these operations, limiting the technique to experts in data analysis and to those with sufficient computational expertise to create the analysis software.

Our group has spent the past 8 years developing DFXM scanning and analysis techniques with complementary theory and simulations. We developed the time-resolved version of DFXM for use on timescales from millisecond to femtosecond. To do this, we established the optical setup to extend DFXM first to time-gated experiments^26^ and then to XFELs capable of capturing pump-probe acquisitions over millisecond to microsecond timescales.^36^ We then established analytical methods to forward model DFXM using analytical theory^30,31,43–45^ and established the artificial intelligence (AI) methods to quantify the image features relevant to our dislocation science.^46–49^

In this article, we review a few of our AI developments and demonstrate how our approaches to develop data science methods then integrate them with theory-enabled insights that are not possible without the physics-informed methodology. We demonstrate this from our work establishing ways to understand time-resolved dislocation dynamics^46,47^ and to dimensionally reduce 4D DFXM data into 3D dislocation structures.^48,49^ We close by introducing complementary developments by other groups and discuss how these strategies may be combined to enable future exciting opportunities across materials science.

## Dislocation dynamics in time-resolved DFXM studies

Our initial DFXM work focused on synchrotron studies of dislocation dynamics—necessitating analysis methods to enable statistical sampling of these dynamics. To accomplish this, we developed a semi-automated workflow, guided by dislocation theory, that allowed us to identify a stabilizing tilt boundary of (110) edge dislocations in single-crystal aluminum.^47^ The semi-automated workflow was designed to identify the positions of all five dislocations (i.e., the “objects of interest”) forming this boundary across a selected 60 frames corresponding to nearly 15 s in real time.

Our approach began with a 2D Stationary Wavelet Transform (SWT) employing a Daubechies-4 orthogonal wavelet, focusing on the 3rd-level horizontal detail coefficients to enhance the bright features indicative of dislocations within the raw DFXM images. These “wavelet-marked” features were binarized by subtracting the median of the array and applying a suitable threshold (3.5 times the standard deviation). We note that the threshold used in this study is dependent on the sample, incident flux, exposure time, and detector. The analysis was then followed by the removal of small objects and a morphological closing operation to refine the bright regions. Noticing that dislocations exist as adjacent bright- and dark-region pairs due to lattice distortions, we noted that the initial SWT segmentation captured only the bright segments. To address this, we used the Fast Marching Method (FMM) for region-based segmentation, seeding it with points derived from the centroids of the bright regions. Specifically, for each bright centroid, we searched a $25\times 25$ pixel neighborhood to locate the minimum intensity pixel, designating it as the seed for segmenting the corresponding dark region. The resulting bright and dark regions were combined into composite dislocation objects, smoothed with a Gaussian filter to eliminate artifacts. Across the 60 frames, we observed instances of overlapping dislocations, which we resolved using a Kalman Filter (KF) assuming linear motion of the strained region for subsequent frames. The KF predicted dislocation positions with a state transition model, and the Munkres assignment algorithm then labeled each dislocation consistently across frames by minimizing the Euclidean distance between KF-predicted and detected centroid positions.

**Fig. 2:**
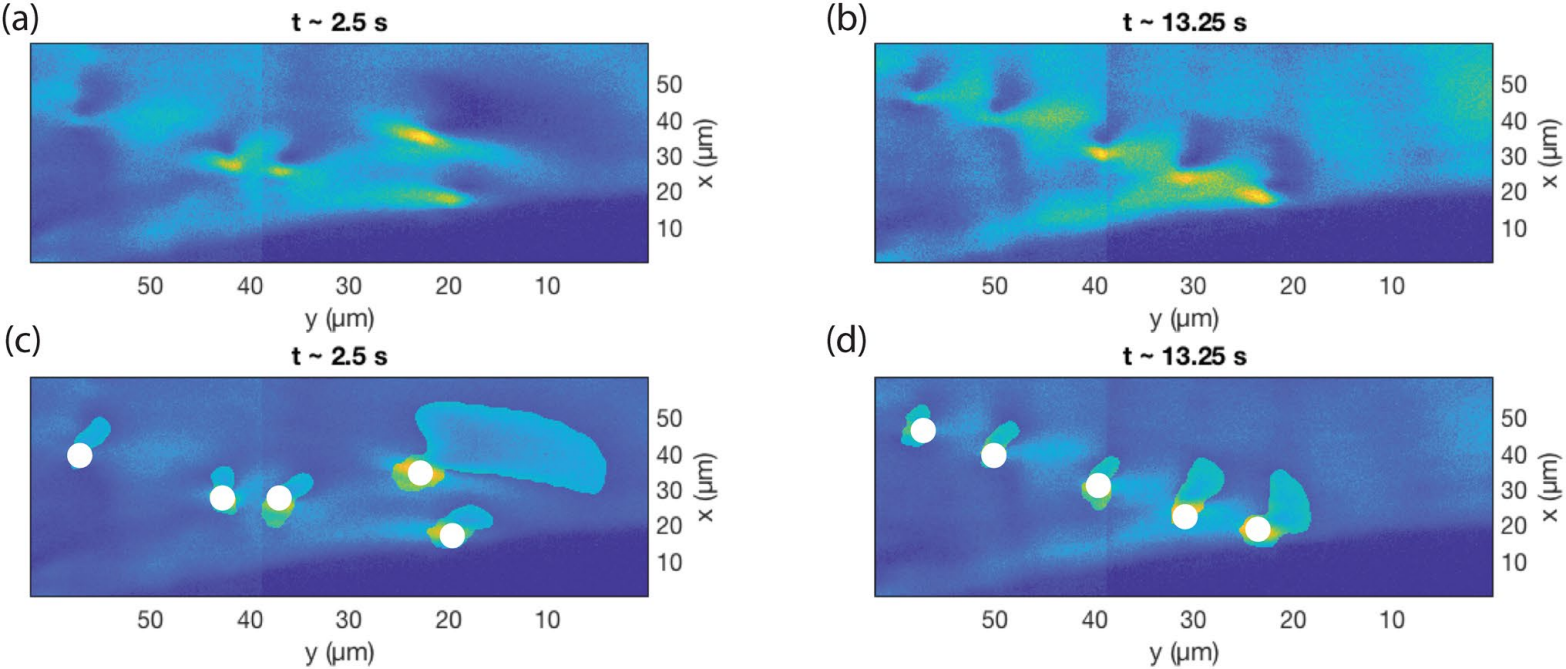
Segmentation of the displacement gradient field of moving dislocations. (a) and (b) Display DFXM image frames where a dislocation move to the dislocation boundary over time. (c) and (d) Demonstrate the position of the dislocation (white circle) and the segmentation of the displacement gradient field produced by these dislocation using the detailed methods outlined in Gonzalez *et al.*^47^ Adapted from Gonzalez *et al.*^47^

This process produced a comprehensive temporal stack of dislocation positions without prior knowledge of their number, and was then used to guide a subsequent study, with manual oversight to avoid misinterpretation.^26^ The resulting dataset in the study on dislocation dynamics was able to resolve the 4-Hz dynamics of 13 dislocations in a single crystal of aluminum ranging from annealing temperatures of 0.97$-$0.99 of the melting temperature. The >10,000 dislocation positions in that work enabled statistical analysis of the motion to inform the thermally activated variance in the RMS position that ultimately led to the conclusions of thermal forces overwhelming the inherent stability of the boundary.

However, a shortcoming of this approach was that it required the deformation field emanating from the dislocation to have the specific shape shown in Fig. 2 to be identified and tracked by the method. To integrate this approach into more versatile experiments of dislocation dynamics, a version of this semi-automated analysis method was required that could be informed by the physics of how DFXM samples the dislocations. To improve the accuracy of dislocation detection and quantification, a Bayesian inference method was used to estimate dislocation positions from DFXM images, which achieved super-resolution accuracy that extends the dislocation position information beyond the optical resolution.^46^

**Fig. 3:**
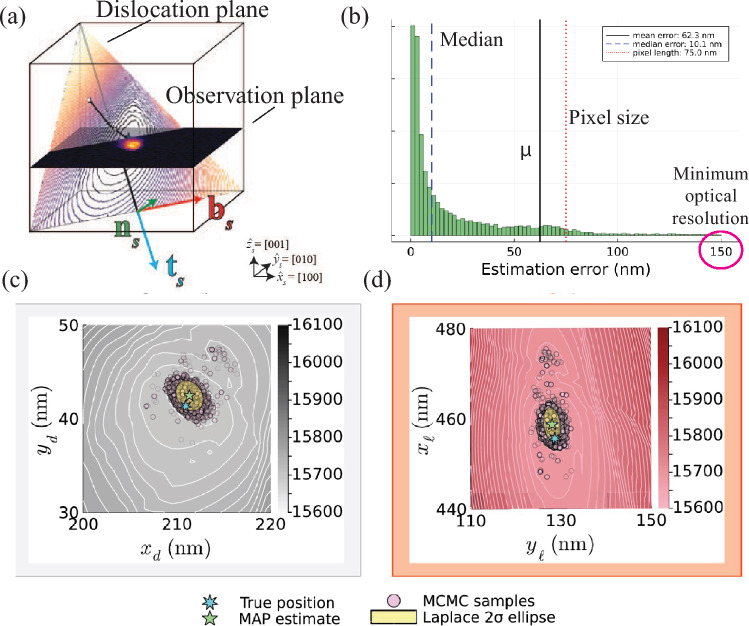
Demonstration of super-resolution dislocation position estimation using Bayesian inference on DFXM images. The method is illustrated through: (a) real space representation of the dislocation and observation plane geometry, (b) position estimation error demonstrating super-resolution capability below the pixel size, (c) comparison of Laplace approximation and MCMC sampling results in the algorithm plane, and (d) corresponding results mapped to the image plane. Adapted and modified from Brennan *et al.*^46^

In this work, Bayesian inference refines the initial dislocation position estimates using a statistical framework based on the integrated physical models of the DFXM contrast and experimental noise.^46^ The method begins by forward modeling the deformation field, denoted ${\mathcal {F}}(\xi )$, to simulate a noise-free DFXM image based on a dislocation position $\xi$ based on the formalism devised in Poulsen *et al.*^30^ This model combines continuum mechanics to describe the displacement gradient field, $\nabla {\textbf{u}}$, around the dislocation, and a ray optics formalism to account for X-ray diffraction and imaging optics. To adapt this to simulated experimental data, a likelihood model incorporates detector noise, treating observed pixel intensities as random variables influenced by the dislocation signal, background counts, and electronic read-out noise. The intensity of each pixel is modeled as a Gaussian distribution with mean $m_{n}(\xi ) = {\mathcal {F}}(\xi )_{n} + {\bar{\beta }}$ (where ${\bar{\beta }}$ is the background count) and variance $\sigma _{n}^{2}(\xi ) = {\mathcal {F}}(\xi )_{n} + {\bar{\beta }} + \sigma ^{2}$, replicating the noise characteristics observed in DFXM experiments (Fig. 3).

The Bayesian framework combines this likelihood with a prior distribution, which encodes initial knowledge or estimates of the position of the dislocation, which is often a uniform distribution over a region informed by image features or prior analysis.^50^ Applying the Bayes rule, the posterior distribution $\pi (\xi \mid {\boldsymbol{I}}^{\text {obs}})$ is derived, showing the probability of dislocation positions given the observed image ${\boldsymbol{I}}^{\text {obs}}$. This posterior enables both point estimation and uncertainty quantification, providing a comprehensive view of dislocation locations that were sampled by three different computational approaches that balance accuracy *vs.* computational cost.

We validated the Bayesian inference method using synthetic DFXM images of edge dislocations in single-crystal aluminum, generated with known ground-truth positions, and achieved an error of approximately 3 nm, which is >10$\times$ smaller than the 75 nm pixel size. Indeed, across the 5000 trials for each of the 12 edge dislocation characters in aluminum of this particular experiment, the median errors were 5.3 nm under low noise conditions (5% background noise, 1% read-out noise relative to maximum intensity) and 14.2 nm under high noise conditions (10% and 5%, respectively). The resulting “super-resolution” capability enables high-fidelity imaging of well-separated dislocations with accuracy comparable to TEM types of experiments in deeply bulk materials systems (e.g., $>100\,\upmu$m beneath the surface). Although this approach has not yet been extended to experimental data, we foresee this as an opportunity for future studies, as discussed further in Sect. 4.

## Dimensionality reduction of DFXM data

Following our initial time-resolved studies on a single observation plane, we collaboratively developed a new 3D dislocation characterization method with DFXM that required a new data science need in DFXM data, i.e., dimensional reduction from the many-axis scans, in our subsequent study.^49^ The resulting 4D dataset, comprising spatial coordinates $(x, y, {\text {and }}z)$ and angular tilt $\phi$, covering a large 3D volume ($100 \times 300 \times 300 \, \upmu {\text {m}}^{3}$), required sophisticated dimensionality reduction techniques to characterize the dislocation boundaries and their governing vectors. Our initial approach involved manually selecting images for each *z* layer that exhibited the weak-beam condition, a state where the main crystal lattice deviates from the Bragg condition but highly strained regions, such as dislocations, diffract X-rays and appear as bright spots in the off Bragg condition. This phenomenon is analogous to weak-beam imaging in TEM, where dislocations are highlighted due to local lattice distortions.^51^ However, the visibility of dislocations in DFXM depends on the 2D angular orientation of the crystal (defined by tilt angles $\phi$ and $\chi$), which determines whether dislocations are visible or invisible in different weak-beam frames. For a detailed derivation of the dislocation invisibility criterion and their sensitivity to different scans in DFXM, we refer the reader to our recent theoretical work by Pal *et al.*^44^ and Kanesalingam *et al.*^31^

In that work, we manually selected a single image corresponding to the optimal weak-beam condition for each *z* layer, and applied a binary thresholding technique to threshold the pixels, identifying those most likely to contain dislocations. These binarized images were stacked along the *z* direction to construct a 3D representation of the strained region, showing several dislocation boundaries within the crystal.

**Fig. 4:**
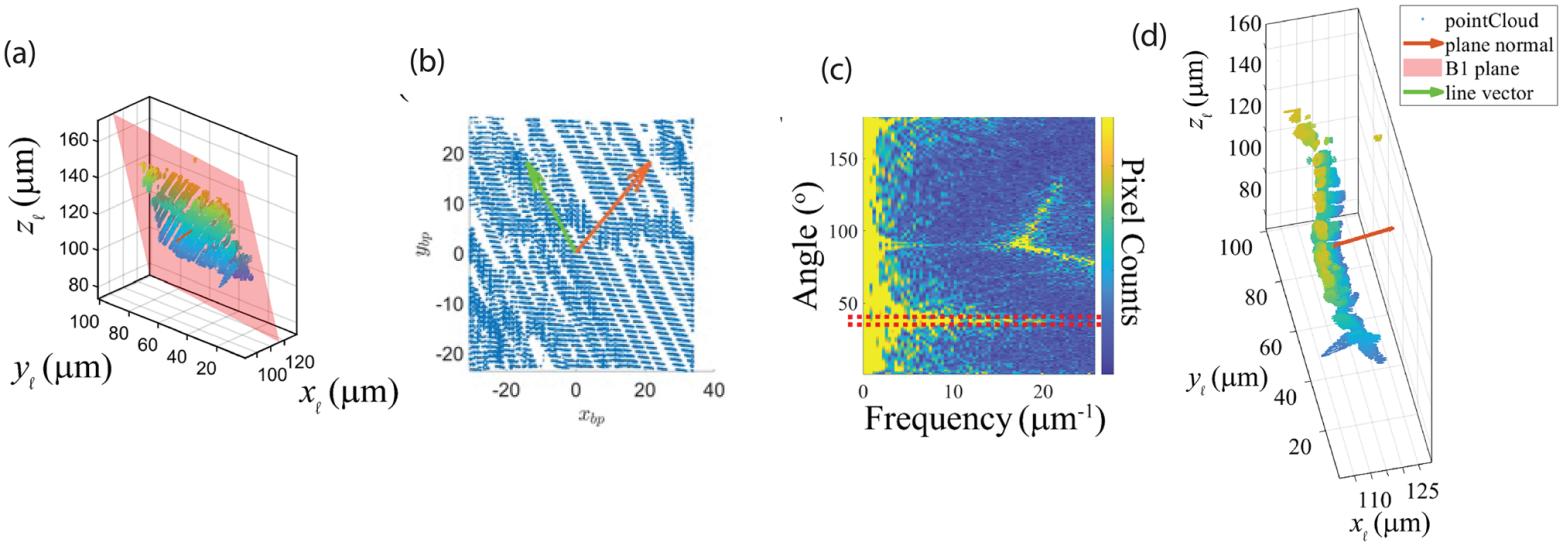
Workflow for MSAC identification of dislocations and their boundaries from $\phi ,z$ DFXM scans. The workflow consists of 5 steps: (a) define the normal vector ${\vec {n}}$ in the real space coordinates (*x*, *y*) denoted by $\mathbb {{\{R}_{\ell} \}}_{(x,y)}$, (b) define the boundary plane coordinates $\mathbb {\{{R}_{\text {bp}}\}}_{(x,y)}$ and the line vector ${\vec {\ell }}$, (c) define the dislocation space $\mathbb {\{R}_{\text {d}}\}_x$ using Fast Fourier Transform (FFT), and (d) transform from dislocation space $\mathbb {\{R}_{\text {d}}\}_x$ to real space coordinates $\mathbb {{\{R}_{\ell} \}}_{(x,y,z)}$ to obtain the tangent vector ${\vec {t}}$. Adapted and modified from Yildirim *et al.*^49^

To quantify the character of those dislocations and associated boundaries, we designed the workflow shown in Fig. 4. Firstly, we represent the dislocation voxels as a 3D point cloud and used the M-estimator SAmple Consensus (MSAC) algorithm^52^ to robustly fit a plane to these points. MSAC, an extension of RANSAC, minimizes a robust cost function (e.g., median of residuals) to handle outliers, voxels that may not belong to the primary boundary. The normal vector ${\vec {n}} = (a, b, c)$ of the fitted plane describes the boundary’s orientation in the crystal. Using ${\vec {n}}$, we transform the coordinate system to align ${\vec {n}}$ with the *z* axis, projecting the 3D dislocation structure onto a 2D plane.

In the 2D projection, the dislocation lines appeared as parallel linear features. To determine their direction, we performed 360 rotations of the 2D image in 1° increments and summed the pixel intensities along the vertical axis for each rotation angle $\alpha$. This projection effectively creates a one-dimensional intensity profile by integrating along one direction of the image. When the integration direction is perpendicular to the linear features, the projection highlights the periodicity of these features with maximum contrast, enabling us to identify the orientation of the dislocation lines.

To quantitatively analyze this periodicity, we used the Fourier transform of the projection profiles. The Fourier transform converts the spatial information into frequency components, highlighting the dominant periodicity in the projected data. By examining how these frequency components vary with the rotation angle, we could identify the angle $\alpha _{0}$ that maximizes either the projection variance or the power at specific frequencies in the transform. This angle indicates the direction perpendicular to the dislocation lines, which means the line vector ${\vec {l}}$ in the 2D plane points along $\alpha _{0} + 90^{\circ}$.

Then, the 2D line vector was transformed back to a 3D coordinate system. This transformation involved expressing the 2D vector in terms of the basis vectors of the plane, which were derived from the normal vector ${\vec {n}}$. The resulting 3D line vector ${\vec {l}}$, along with ${\vec {n}}$, was expressed in the original crystallographic coordinate system. With ${\vec {n}}$ and ${\vec {l}}$ determined, we applied Frank’s rule to estimate the Burgers vector ${\vec {b}}$. For a tilt boundary, there is a simple relationship between the angle of misorientation $\theta$, the spacing of dislocation *d*, and the magnitude of the Burgers vector *b*: the angle of misorientation equals the ratio of the magnitude of the Burgers vector to the spacing of dislocation. This relationship is fundamental in dislocation theory and allows us to verify our measurements by comparing calculated and observed values.

Our analysis method in this work ultimately allowed us to use DFXM to characterize self-organized dislocation structures within a millimeter-scale aluminum single crystal after isothermal annealing at 590° C for 10 h. Assuming a predominant tilt character, ${\vec {b}}$ is perpendicular to ${\vec {l}}$ and lies in the plane, often approximated as ${\vec {b}} \parallel {\vec {n}} \times {\vec {l}}$ (e.g., ${\vec {b}} = [110]$ for B1). The measured misorientation angles (e.g., $0.0418 \times 10^{-5}$ rad for B1) corroborated the calculated spacings. This approach effectively characterized dislocation boundaries, revealing a triple junction (B1–B1$'$–B1$''$) with a 48°, angle deviating from the 120°, predicted by conventional annealing theory suggesting additional stabilization mechanisms, possibly due to pinned dislocations or impurities.

The impact of our initial methods has been significant, as many experiments have since benefited from dimensional reduction of 4D scanning datasets. However, we note that manual selection of a single weak-beam condition for each observation plane has been highly limiting. For well-defined single crystals, this imposed a time-consuming manual aspect of the analysis that limited the applicability of high-dimensional scans to guide DFXM experiments. Furthermore, many DFXM datasets had no single weak-beam frame because different subgrains had different optimal weak-beam conditions because the dislocation boundaries surrounding them caused local orientation differences. As such, a single frame is insufficient to describe a weak-beam condition for the entire 4D dataset.

**Fig. 5:**
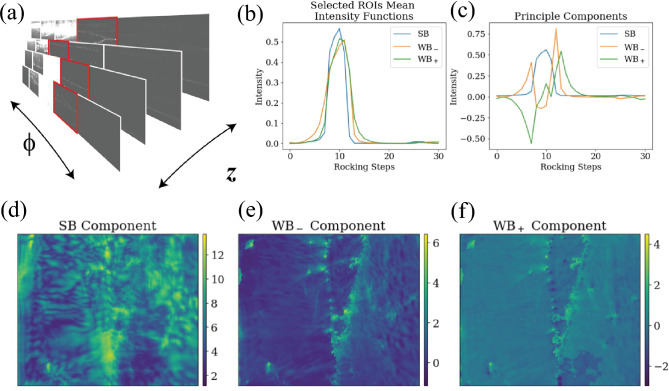
Gram–Schmidt orthogonalization framework for dimensionality reduction for DFXM data. (a) 4D scan of a single crystal aluminum sample showing $\phi$ and *z* scans. The subsequent analysis focused on a single observation plane (single *z*). (b) Mean intensity profiles of selected regions of interest (ROIs) showing strong-beam and weak-beam conditions across different rocking angles. (c) Principal components extracted from the ROIs using Gram–Schmidt orthogonalization. (d–f) Resulting orthogonalized DFXM image components showing the separation of strong-beam and weak-beam signals. Adapted and modified from Huang *et al.*^48^

To generalize the selection of weak-beam conditions and manage the large volume of DFXM data, we therefore developed a relatively unsupervised method using Gram–Schmidt orthogonalization^53^ in the subsequent work of Huang *et al.*,^48^ as shown in Fig. 5. Compared to Principal Component Analysis (PCA),^54^ a widely used dimensionality reduction technique that identifies directions of maximum variance, this Gram–Schmidt-based approach incorporates physical priors through ROI selection. The data-driven components of PCA lack inherent crystallographic meaning, while the strong-beam and weak-beam components directly correspond to diffraction conditions critical to dislocation studies. Furthermore, enforced orthogonality simplifies coefficient interpretation, offering a clearer separation of signals compared to PCA’s potentially correlated components. Gram–Schmidt orthogonalization decomposes a 3D DFXM dataset (*x*, *y* and $\phi$) into three physically significant components, corresponding to the contrast generated by the deformation gradient field, at different $\phi$ values.^48^ Strong-beam condition (SB) represents the undeformed crystal domain with high intensity dynamical diffraction fringes, while the weak-beam conditions (${\textrm{WB}}_{-}$, ${\textrm{WB}}_{+}$) capture dislocation-specific features on either side of the rocking curve, revealing anomalous lattice distortions.

Gram-Schmidt orthogonalization process ensures that $u_{\text {SB}}, u_{\text {WB}_{-}}, u_{\text {WB}_{+}}$ are mutually orthogonal, eliminating overlap between components. The method begins with dataset preprocessing, including logarithmic scaling and offset correction, to enhance dislocation visibility. Then three ROIs are manually selected to define basis functions for SB, ${\textrm{WB}}_{-}$ and ${\textrm{WB}}_{+}$ to capture the dislocation-specific features. However, manual ROI selection introduces subjectivity, suggesting potential improvements through automated clustering or machine learning for scalability to complex datasets. The preprocessed intensity profile $I'(x, y, \phi )$ is then projected onto the orthogonal basis with the corresponding coefficients. These coefficients $c_{k}(x, y)$ form three 2D images, each representing the contribution of the corresponding component to the sample.

Orthogonalization ensures that the selected components are independent, reducing the 31 $\phi$ resolved stack (3D) into three 2D images, significantly decreasing the data size by nearly 90% while preserving the dislocation information essential for characterization. Furthermore, the strong-beam component isolates dynamical diffraction effects, while the weak-beam components contain the necessary dislocation features for subsequent analysis. Our paper applied this method to a pristine aluminum single crystal and successfully separated strong and weak-beam signals, effectively isolating dislocation signatures even in a multi-subgrain system.

## Discussion and outlook

From identifying the dislocation dynamics to dimensionality reduction in large volume DFXM scans, our analytical approaches have shown how data science methodologies can meaningfully advance DFXM analysis and, more importantly, how their integration with physical models yields unprecedented gains that could not have been achieved without AI implementation. Our analytical approaches progressed from a semi-automated workflow using wavelet transforms and Kalman filtering for temporal tracking of dislocations, to a Bayesian inference framework achieving “super-resolution” position determination of dislocation cores, and finally to Gram–Schmidt orthogonalization that decomposed 3D datasets into physically meaningful components corresponding to strong and weak-beam conditions in diffraction. Throughout these developments, dislocation theory, including Frank’s rule and weak-beam principles, provided essential physical constraints that guide our approaches.

Other groups are now starting to advance the use of AI in DFXM analysis. In particular, Garriga Ferrer *et al.*^32^ has developed the *darfix* package, which implements blind source separation (BSS) techniques for the images collected with DFXM. BSS is a widely used unsupervised learning algorithm that can disentangle mixed signals into their constituent components with minimal prior knowledge about the source signals or the mixing process.^55^ *darfix* package includes techniques such as PCA, nonnegative independent component analysis (NICA), and nonnegative matrix factorization (NMF) to decompose overlapping diffraction signals from the crystalline domains. Another notable study by Abulshohoud *et al.*^56^ has demonstrated multiresolution analysis using wavelet transforms (Haar and Daubechies wavelets) to extract mesoscale characteristics such as the tracking of twin boundaries. Their approach effectively separates illumination profiles from edge features across multiple lengthscales.

These works represent important foundational contributions to the advancement of automated DFXM analysis. Both studies use data science approaches; however, we point out the opportunity to incorporate the theory of image formation into these representations, making them more interpretable. The integration of physical constraints, as demonstrated in our work, provides advantages in terms of robustness and meaningful feature extraction. Future opportunities include physics-informed neural networks,^57^ convolutional architectures for automated feature detection,^58,59^ and uncertainty quantification methods^60^ that could transform DFXM from a largely interpretive technique into a predictive materials characterization tool capable of real-time microstructural evolution forecasting.

Toward the possibilities of incorporating physical interpretations into DFXM analysis, we highlight several theory and simulation works published in recent years that could potentially fuel the integration of physics-informed models into DFXM. For instance, Pal *et al.*^44^ formulated a theoretical model that adapts the “invisibility criteria” from TEM to DFXM, enabling the direct measurement of a dislocation’s Burgers vector from 2D rocking curves obtained with a single peak. Similar work by Borgi *et al.*^61,62^ has also explored the identification of dislocations with DFXM, including the simulation and classification of dislocations using a covariance matrix. This work helps to connect DFXM observations to fundamental dislocation theory and could enable significant advances if extended to high-throughput methods for fields studying isolated dislocation structures.

Further simulation advances have focused on high-dislocation density systems^45^ for which DFXM signals are more difficult to interpret at the single-dislocation level. Wang *et al.* developed a scalable forward model, DD-DFXM, which computes virtual DFXM images from complex discrete dislocation structures derived from large-scale atomistic simulations. This work could aid in the interpretation of DFXM images of high-dislocation–density systems relevant to crystal plasticity.

Considering the reconstruction of the full deformation gradient tensor from DFXM scans, Detlefs *et al.*^63^ introduced oblique diffraction geometry that could facilitate scanning of noncoplanar reflections with the same illumination volume. Based on this work, Kanesalingam *et al.*^31^ developed an inverse modeling formalism to reconstruct the full tensor of the deformation gradient, and subsequently the strain and lattice rotation, from the DFXM observables. This computational framework includes forward calculation of deflection angles and sensitivity analysis, which assists both in the interpretation of experiments and the design of optimal DFXM measurement strategies, extending to finite-deformation scenarios. Henningsson *et al.*^64^ introduced a regression framework to reconstruct full deformation gradient tensor fields from DFXM data under kinematic diffraction assumptions following the oblique diffraction geometry. Their work allows for the derivation of local dislocation densities and shows promise for directly interfacing DFXM measurements with dislocation dynamics models.

These collective advances in theory and simulation are paving the way for more physically grounded and quantitative interpretations of the DFXM data. In addition, these works provide essential physical models and simulation frameworks that enable a more sophisticated integration of machine learning approaches into the DFXM.

The adoption of AI methodologies has shown transformative capabilities in many X-ray and electron microscopy studies. Specifically, Physics-Informed Machine Learning approaches have successfully addressed fundamental microscopy challenges. These include ill-posed inverse problems in CT reconstruction,^65,66^ phase retrieval in X-ray ptychography,^67^ and low signal-to-noise ratios in cryo-EM.^68^ These problems are overcome by systematically integrating physical constraints such as the Beer–Lambert law, diffraction theory, and electron scattering physics^65,67,69,70^ directly into neural network architectures and loss functions. This physics-informed approach delivers improved data efficiency, robustness, and generalization compared to purely data-driven methods, as demonstrated in various modalities from automated STEM aberration correction^71^ to cryo-EM synthetic data generation.^68^ These successes validate that the incorporation of domain-specific physical knowledge transforms AI from statistical pattern recognition into scientifically grounded analysis tools.^72^

The integration of AI with physics-based models has fundamentally improved the analytical capabilities of DFXM, creating unprecedented opportunities for quantitative microstructural characterization. Our methodological developments demonstrate that AI-enhanced analysis achieves results that would not have been possible without AI, providing essentially a new approach when guided by established physical principles rather than operating through purely data-driven approaches. We see an opportunity for the emerging data science approaches in DFXM to continue finding opportunities to link with physical models and DFXM simulation tools for predictive modeling capabilities. We note that this type of physics-informed data science approach can be a starting point for autonomous experimentation, which is an up-and-coming direction for the field. Although our group has begun exploring this direction (for example,^73^ and^74^), this is outside the scope of this work.

As DFXM theory matures and AI-driven interpretation methods advance, new opportunities emerge to connect DFXM experiments to real-world applications including defects in semiconductors, high-dislocation–density systems, battery materials, and thermal electronic engineering. The physics-informed approaches that we have developed, combined with emerging simulation frameworks, position DFXM to transition from a characterization tool to a predictive instrument for materials design. AI-enhanced DFXM analysis may become essential to make its complex analysis accessible to the broader materials science community.

## Data Availability

No datasets were generated or analyzed during the current study.
